# “Choosing the main character”: healthcare professionals’ attitudes towards counselling patients about risk disclosure to relatives in the era of mainstream cancer genetic testing

**DOI:** 10.1007/s10689-025-00516-1

**Published:** 2025-12-12

**Authors:** Anna Öfverholm, Matilda Liljedahl, Agnes Elmberger, Per Karlsson, Anna Rosén

**Affiliations:** 1 Department of Oncology, Institute of Clinical Sciences, Sahlgrenska Academy, Gothenburg University, Gothenburg, Sweden; 2 Department of Clinical Genetics, Sahlgrenska University Hospital, Gothenburg, Sweden; 3 Department of Oncology, Sahlgrenska University Hospital, Gothenburg, Sweden; 4 Department of Learning, Informatics, Management and Ethics, Karolinska Institutet, Stockholm, Sweden; 5 Department of Diagnostics and Intervention, Umeå University, Umeå, Sweden

**Keywords:** Hereditary cancer risk, Genetic counselling, Mainstream testing, Risk disclosure

## Abstract

**Purpose:**

This study explored healthcare professionals’ attitudes toward counselling patients on disclosing genetic cancer risk to relatives. Genetic counselling practices in Sweden are undergoing significant changes due to the increased use of genetic testing to assess hereditary risk and the implementation of mainstreamed testing in oncology care. These developments require an assessment of healthcare professionals’ perceived roles and responsibilities when working with patients with hereditary risk. Best practices need to be developed to effectively support risk disclosure, and subsequently risk management, to relatives.

**Method:**

Data was collected through interviews with oncologists, gynaecologists, surgeons, clinical geneticists, and genetic counsellors, working in oncology care or at cancer genetics units. Data was analysed using reflective thematic analysis.

**Results:**

The results are presented as four positions that healthcare professionals take, describing their attitudes towards counselling patients about risk disclosure to relatives. The position depends on whether they perceive healthcare or the patient as ultimately responsible for risk information reaching relatives, and whether their focus is on the patient or the at-risk relatives. There are several stakeholders involved, and hence 'characters in play'.

**Conclusion:**

These results could serve as a basis for discussions on roles and responsibilities, while developing best practices regarding genetic counselling on hereditary cancer risk communication.

**Supplementary Information:**

The online version contains supplementary material available at 10.1007/s10689-025-00516-1.

## Introduction

Positive genetic test results from screening cancer predisposition genes have implications for the patient, and the possibility of subsequent cascade testing has implications for at-risk relatives (ARRs). Predictive testing of cancer-free individuals for a familial variant with clinically relevant risk enables access to targeted programs for early detection and prevention of cancer [1, 2], which is one of the major gains in the growing field of genomic medicine.

With genetic testing comes the need for genetic counselling, described as the communication process of helping individuals understand and adapt to the medical, psychological, and familial implications of genetic risk [3, 4]. A central aspect of genetic counselling is the topic of family-mediated risk disclosure. This refers to the shared responsibility between the healthcare professional (HCP) and the patient, where the HCP is responsible for informing the patient about hereditary risk, and the patient is responsible for disclosing information to their ARRs [5]. Guidelines from different countries share common recommendations; HCPs should encourage patients to disseminate information and offer support to patients in the process [6, 7]. The responsibility of HCPs towards relatives could be described as a 'duty to care', that is, an obligation on the part of the healthcare system to inform and assist the public in preventing serious and avoidable health risks. It co-exists with the fundamental principle of patient confidentiality [8]. The patient’s 'duty to share' could be defined as a moral duty, with hereditary risk being familial in nature [9, 10]. However, existing working models for supportive and structured handling of patients’ disclosure to their families have limitations, and the practice is multifaceted and complicated [11–13]. In Sweden as well as other European countries, Australia and USA, working models including genetic testing and counselling are changing as the demand for genetic assessments is increasing. Mainstreamed genetic testing has been partially implemented within the Swedish healthcare system. Non-genetic HCPs**,** such as oncologists, surgeons and gynaecologists, now offer genetic testing as a part of the diagnostic and treatment processes for breast, ovarian and colorectal cancer [14]. An ongoing discussion in healthcare and research is how to articulate the responsibility for ARRs autonomy and agency regarding hereditary risk, both in practical guidelines and in legislation [11, 15, 16]. This responsibility is a topic of discussion in cancer genetics communities in many countries and not yet formulated in Swedish national guidelines.

As the established working models for genetic testing and counselling are changing, there is a growing need to reassess the roles and responsibilities regarding when and how information is communicated to patients and disseminated to their families. We need to understand more about the thoughts, feelings, and actions, of both non-genetic and genetic HCPs in pre- and post-test counselling, when handling cancer genetic testing and the issue of risk disclosure. The aim of this study was to explore healthcare professionals’ attitudes towards the practice of counselling patients about risk disclosure to relatives at risk.

## Participants and Methods

### Setting

This qualitative interview study included non-genetic and genetic HCPs in Sweden, who interact with patients with cancer undergoing screening of cancer susceptibility genes, healthy relatives undergoing predictive testing for a familial variant, and/or individuals in targeted risk management. Local routines and regional healthcare organisation in Sweden differ; pre-test information is provided to patients by either non-genetic HCPs in mainstream testing at oncology care units, by genetic HCPs at cancer genetics units, or by genetic or non-genetic HCPs at combined units. A patient with a positive genetic screening is offered post-test counselling with a genetic or non-genetic HCP at a cancer genetics unit or a combined unit, ARRs are offered pre-test and post-test information and counselling at these same units. The most common diagnosis among these patients is hereditary breast- and ovarian cancer and Lynch syndrome.

### Method

An interview guide (supplementary info A) was developed based on the research question, existing literature, and the authors’ expertise in cancer genetics, undergoing minor revisions during data collection. Individual, semi structured interviews were conducted using the interview guide as a flexible framework. Three authors (A.Ö., M.L., A.E.) and a research assistant (C.N.) took turns in interviewing participants to ensure that the interviewer had not been colleague of the HCP. Interviews were conducted either in person (n = 10) or remotely via video link (n = 4) between July 2022 and March 2024. The interviews lasted 22–46 min (median 36 min), were audio-recorded and transcribed verbatim. Participants’ attitudes were defined as a composite of three aspects: cognition (i.e., thoughts and beliefs on a phenomenon), affect (i.e., feelings evoked by the phenomenon), and behaviours (i.e., the influence on actions) [17]. We therefore sought to investigate healthcare professionals’ thoughts and feelings associated with counselling patients about risk disclosure, and how they approached this in practice.

### Participants

We aimed for maximum variation sampling [18], including HCPs with different clinical backgrounds and from different hospitals. Eligible HCPs were identified through the professional network of AÖ and AR. In total, 20 HCPs from clinical genetics units, oncology units and combined units were invited by email, none declined participation, but six HCPs did not respond though they were sent two separate reminders. Fourteen HCPs agreed to participate and were included in the study (Table 1). Four participants were genetic HCPs (genetic counsellors or clinical geneticists) whereas ten participants were non-genetic HCPs (oncologists, breast surgeons or gynaecologists). These participants, 13 women and one man, represented both university hospitals and regional hospitals from three out of six health care regions in Sweden. The participants’ practice ranged from providing basic pre- and post- test information about treatment-informing genetic testing to thorough pre- and post-test counselling to both patients with cancer and healthy individuals. Participants’ encounters with patients occurred either on a few occasions or through recurring consultations in long-term care.

**Table 1:** Participants’ demographics

Participants’ demographics	n
Women/men	13 / 1
Working experience, full- or part time	3.5–24 years
*Age*	39–66 years
*Professional background*	
Genetic HPs: clinical geneticist, genetic counsellor	4
Non-genetic HPs: oncologist, gynaecologist, surgeon	10
*Main workplace*	
Oncology care	5
Cancer genetics unit	6
Combined oncology care, risk management-, and cancer genetics unit	3

### Data analysis

Data were analysed with reflexive thematic analysis, conducted iteratively with data collection [19, 20]. The interview transcripts were reviewed and meaning units were identified and organised into preliminary categories. These categories were later refined, and themes, later called positions, were identified. The analysis was led by A.Ö. and M.L., but all authors took part in regular readings and discussions. The research group comprised clinician researchers with experience from clinical cancer genetics and counselling, four of the authors were proficient in qualitative research methodology.

## Results

**Fig. 1:**
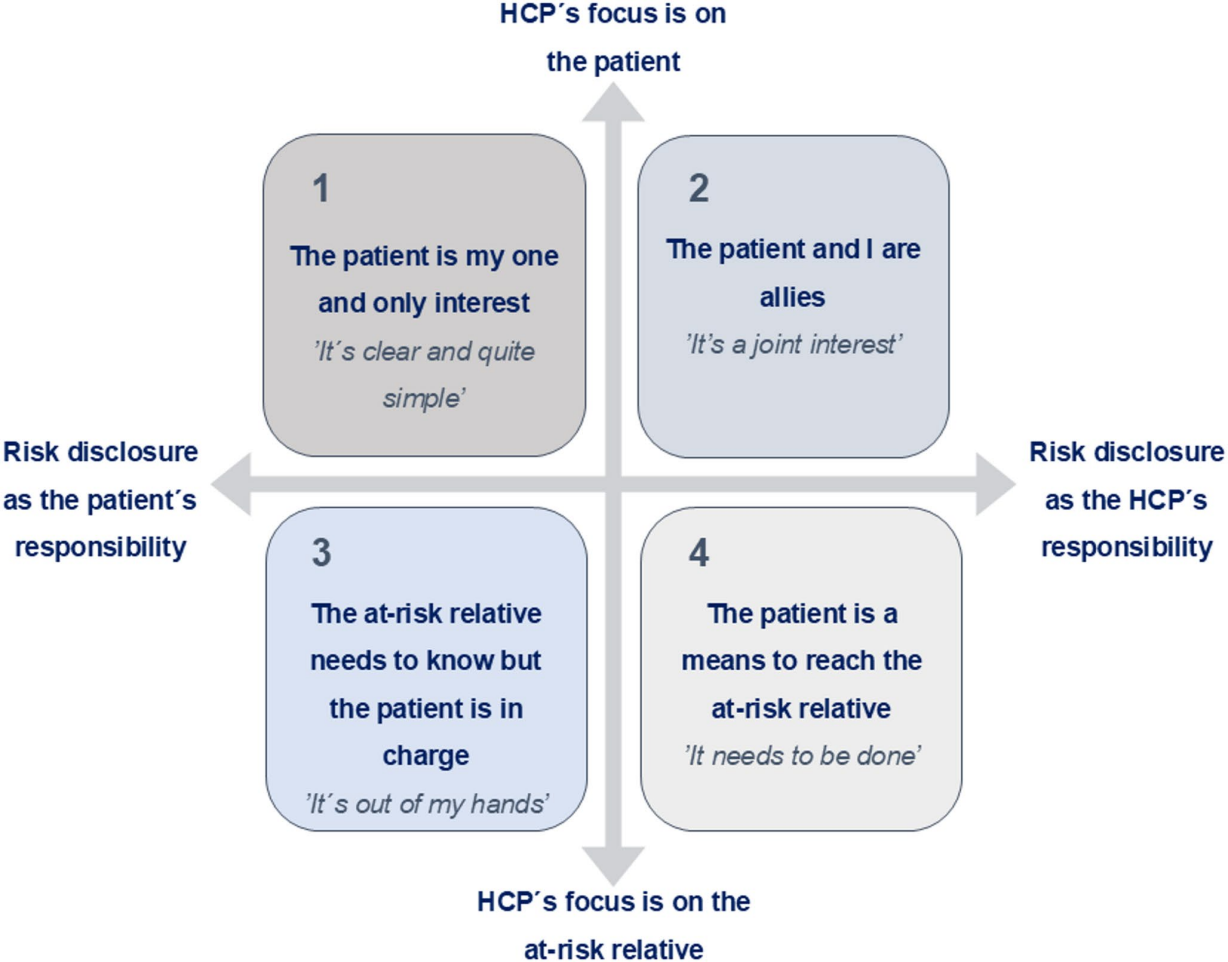
Four positions describing HCP’s attitudes towards counselling patients about hereditary cancer risk disclosure to relatives. The standpoints are shown on a spectrum: the Y-axis is about who the HCP has in focus, and the X-axis is about how the HCP perceives the responsibility for relatives being informed. The positions are phrased as statements and shaped by the HCP’s profession, workplace and assignment but also by the perception of the patient’s health status and knowledge about relatives. HCPs described how they have, take, or move between these positions

Four positions were developed reflecting HCPs’ attitudes on counselling patients about hereditary cancer risk disclosure to relatives, visualised in Fig. 1. The positions illustrate different ways in which an HCP can approach counselling the patient and are distinct from one another based on variations in participants’ perspectives on risk disclosure, whether the responsibility lies ultimately with the patient or the HCP, and whether the HCP's focus during the consultation is on the current patient or ARRs.

A HCP’s position was partly dependent on their background and current workplace, as well as the clinical situation such as their perception of the patient's current health status, the family tree, and the characteristics of the cancer risk. This meant that a HCP could move between positions and hence counsel a patient regarding hereditary risk in various ways depending on the specific situation. The four positions should therefore not be understood as a fixed state of an individual HCP, profession or workplace but as fluid and situational. A general impression from the interviews was that the participants found their practice relatively well established and felt satisfied in managing their part of the patient’s genetic assessment. However, most of them had experienced recurrent situations, which they perceived as ethically complex.

### Position 1: The patient is my one and only interest: ‘it´s clear and quite simple’

In this position, the focus of the HCP was solely on the patient. The HCP provided basic pre-test information or post-test counselling to the patient but did not explain explicitly in dialogue the relevance for ARRs or explore the patient’s understanding of their responsibility but found it was sufficient to have provided information. In that sense, HCPs in this position had few doubts about their roles – the HCP was not responsible for ARRs, and it was up to the patient to decide if and how to share information. Counselling was hence perceived as ‘relatively easy’.

"I don't see it as my responsibility that potentially affected relatives undergo genetic testing, and it’s actually not my responsibility that they are informed about it either. It's actually the responsibility of the person in front of me; the responsibility lies with them. However, a lot of the responsibility for providing information [to the patient] does fall on me." (Participant no 11: Non-genetic HCP at a cancer genetics unit).

HCPs in this position took the patient’s perspective only, referring solely to the patient’s situation. HCPs in oncology care could refer to prioritising the patient’s medical situation, and that the consequences for ARRs were outside the scope of their assignment or expertise.

"We inform [the patient] that 'there was an error in a gene, but I can't really say if it affects you or not, but we will refer you to the cancer genetics unit’. That's what I say." (Participant no 8: Non-genetic HCP at an oncology care unit).

HCPs experienced in genetic counselling also adopted this position, being committed to the idea that the patient is solely responsible for ARRs. In-depth conversations about ARRs were not seen as their task, they viewed themselves as being neutral in relation to ARRs. HCPs could also acknowledge that there is an important relational aspect to family disclosure, emphasising that the patient probably needed to choose if and how to disclose risk in a considerate way. Another aspect was doubt about the extent to which healthy individuals benefit from predictive testing, which reinforced the idea that HCPs did not have a medical responsibility for disclosure.

I think ultimately, it's up to each person’s own judgment regarding what they want to know… I believe that as a healthcare unit, our role is to simply provide advice and support on how to obtain this information… But I can't quite say it's our ultimate responsibility to ensure it's disseminated. (Participant no 6: Non-genetic HCP at a cancer genetics unit).

### Position 2: The patient and i are allies: ‘it´s a joint interest’

In this position, which was commonly described, HCPs focused on their relationship as a caregiver to the patient but were also significantly engaged in the interest of ARRs.

” I am an oncologist, and … should only do that stuff. […]. And sometimes I have a patient where we found a genetic variant, and then all daughters had the same variant and had to go through the same process. Of course, it matters to the patient. I do think they are grateful that I ask about family members, how it went, and it matters to our relationship, also, I’d say.” (Participant no 5: Non-genetic HCP working at a cancer genetics unit and an oncology unit).

HCPs carefully considered how to best counsel the patient to be able to disclose risk to ARRs, including education about hereditary risk, assessment of the family tree and mutual exploring the relational aspects of ‘bringing bad news’.

“One can use general terms when speaking, like 'my experience tells me that in your situation, there are quite a few who feel this way or think this way.' This allows the patient to relate and perhaps open up to share their own thoughts and feelings. Sometimes, [the patient] can also feel shame, thinking, 'I should understand that it's not my fault [carrying a genetic variant], and I do, but still, I feel this way.' […] One can mention that we all carry different predispositions.” (Participant no 13, genetic HCP at a cancer genetics unit).

For HCPs in this position, patient autonomy and confidentiality were acknowledged, but at the same time they emphasised a professional stance that ARRs’ autonomy and agency should be guarded as well. They described building an alliance with the patient where disclosure to ARRs was a shared responsibility. If the patient was incapable of sharing information for any reason, HCPs tried easing the burden by postponing discussions about ARRs to a later consultation or by presenting alternative courses of action, such as discussing if another family member could help with disseminating information.

“It may be the case that the person themselves is dealing with a disease, undergoing treatment, and having a really tough time. They might not have much energy left, so I sometimes ask if they have a close relative, one who can help with spreading information, someone who is healthy and has a bit more resources at the moment. (Participant no 13, genetic HCP at a cancer genetics unit).

### Position 3: The at-risk relative needs to know, but the patient is in charge:’it´s out of my hands’

In this position, HCPs primarily considered ARRs need to be informed but understood disclosure as a task solely reserved for the patient. This often reflected a practical view of the responsibility of HCPs – if reaching ARRs had been an obligation, there would have been working models and resources. This 'hands-off' position could be described as static, with HCPs expressing feelings of resignation or an attitude of having the right to let go; a combination of duty of confidentiality, lack of formal duty, and the acknowledgement of the importance of the relational aspects of sharing hereditary information.

.…it depends on whether [the patient] is…, who this patient at the Clinical genetic unit […] is, how energetic or how caring he or she is. There is an inequality, or what to say, an unnecessary coincidence perhaps, that determines the outcome [for ARRs]. (Participant no 2, non-genetic HCP at both a cancer genetic unit and in oncology care).

Here, the patient was perceived as being in charge, and the HCP acknowledged the tension caused by the patient’s choice of whether and how to disclose for ARRs to access risk control.

"Imagine, if you sat there [diagnosed with breast cancer], as a sister or a cousin and… 'Darn, they didn't inform me… even though they did know I existed,' sort of. One has to think about this aspect and […] it has started to gain a bit more attention … it's a new era." (Participant no 5: Non-genetic HCP at a oncology care unit).

Further, non-genetic HCPs could consider initiating genetic testing in oncology care with the sole purpose of assessing heredity, but considering the patient’s wants or needs, where pushing for a DNA sample would not be the correct course of action. They could consider bringing up the issue of heredity with a relative accompanying the patient but not contacting other relatives who were not present.

### Position 4: The patient is a means to reach the at-risk relative: ‘it needs to be done’

In this position, HCPs focused on making sure the information was reaching ARRs. Their rationale was mainly the standpoint that carriers of genetic risk who could benefit from targeted risk programs should have the possibility to act upon it. HCPs’ expertise on risk was translated to a sense of duty, in some cases even a right, to warn ARRs. The patient could be seen as a means to reach ARRs. This duty was acknowledged by HCPs as not yet defined in guidelines, and direct contact was considered a future possibility.

“It is w who bear the greatest responsibility because we also understand how inheritance works […]. Even if someone claims to understand, they might have misunderstood something entirely. So, we can't rely on that. We know where the risks lie […]. Hence, we are aware of the risks and possess the expertise on what can be offered [risk management]. (Participant no 1, genetic HCP at a cancer genetics unit).

Moral nudging was described – telling the patient that disclosing information was the right thing to do or mentioning non-disclosure and its consequences if a relative was later diagnosed with cancer. A practical nudge could be handing over letters addressed to the family or contact info to be passed on to relatives. HCPs could suggest to the patient to identify relatives who could assist with risk disclosure, not to ease the burden, but rather to find ways to bypass the patient.

“[Many cancer patients] … struggle and endure and do what we tell them to do without saying that it's hard… So, I wish … that we were the ones who do it [disclosure to relatives]. That they can just let it go and feel that "now you [the HCP] take responsibility, you have found this and you know that they [relatives] benefit from it". I also think it’s natural that it should be our responsibility and not a private individual’s. (Participant no 2: non-genetic HCP with experience from cancer genetics assessments and oncology care).

Genetic HCPs commented on an ongoing discussion in the community about establishing direct contact with ARRs in selected situations. They also described the conflict between patient confidentiality and recurring situations of knowing about several ARRs – having to find a way to preserve confidentiality but also seeing to the interest of all parties being adequately assessed.

## Discussion

In this study we interviewed non-genetic and genetic healthcare professionals in oncology care and cancer genetics services in Sweden, exploring their attitudes towards counselling patients about the disclosure of hereditary cancer risk to relatives. We chose an insider perspective on the issue where we as clinicians aimed to explore the HCP’s attitudes within their existing consultations and thereby move beyond formalised assignments and working models. Given the diversity in participants’ practices we believe it is reasonable to consider these results as representing various ways genetic counselling is practiced in a Swedish setting.

We present the results using a model with four positions, shown in Fig. 1. The participants’ attitudes can be presented on a spectrum in terms of their primary focus – on the patient or the relatives, and their view of who holds the primary responsibility for ARRs – the patient or the HCP, effectively 'choosing the main character' in the consultation.

Several other studies have explored clinicians’ recognition of responsibility for ARRs on an overall level, the extent of this responsibility and the following practices is a subject of division and varies with regulations and speciality [9, 11, 13, 21]. In a qualitative interview study with English HCPs in oncology mainstream testing, the authors suggested a need for a clear and mutual strategy to enable a good practice [15]. A recent study on Portuguese HCPs’ perceptions on roles and responsibilities regarding risk disclosure, describes a balancing act between rights and needs of the patient and the relatives [12].

Delving into the positions, in positions 1 and 2 the HCP had the patient in mind but a varying attitude to the responsibility. Position 1 is patient centred as the HCP did not express a substantial responsibility for the ARR, neither dependency on the patient’s ability to share information. Participants in this position considered a clear message to the patient as being a sufficient effort. In position 2, a well-established position for genetic HCPs, they strived for alliance and a dialogue around the ARRs. This position resonates with a family centred counselling model aiming at reaching and helping the different family members to understand the impact of genetic information in their own life cycle, touching topics such as family structure and dynamics, health beliefs and cultural norms [22, 23].

In positions 3 and 4, the HCP had the ARRs in mind and a varying perception about responsibility as the HCP felt dependent on the patient to reach ARRs and expressed a wish to be more active and to lift the burden from the patient. These positions resonate with findings from a Dutch qualitative study on patients and ARRs in cardio genetics. The authors conclude that considering HCPs to take a more active role could decrease the dependency and the burden on patients to disclose [24].

In the positions 1, 2 and 3, the principle of non-directiveness in genetic counselling was present, albeit not explicitly articulated by the HCP. In position 4, the HCP may consider being more directive, thereby pushing the boundaries. Non-directiveness is an ethically grounded approach that aims not to guide the patient to an outcome predetermined by the counsellor or the genetics service but instead to support the patient in reaching their own decisions. In clinical practice regular reflection on this principle is needed as there can be varying meanings attributed to the concept, something that is described and debated in the literature [25, 26].

Further, in position 2, 3 and 4, HCPs mentioned initiating direct contact with ARRs and reflected on the complexities involved. This reasoning aligns with a recent study from Belgium [11] which identified a tension between HCP´s perceived duty and the constraints of legal frameworks. Consistent with the Belgian findings, participants in position 2, 3 and 4 elaborated on the practical limitations of this duty and the drawbacks of approaching ARRs without a prior personal knowledge of the individual. In line with studies from other countries, participants could also reflect on the need for resources that match the responsibilities assigned [11, 27].

An interesting observation was how non-genetic HCPs commented on how their long term relationships with patients provides opportunity to come back to the longitudinal process of communication within families [28]. It was also considered important to refer patients to genetic HCPs at cancer genetic unit as they are more trained in counselling families. Taken together, a close collaboration between non-genetic and genetic units is important in further developing mainstream testing. 

In line with the qualitative method chosen, we have strived to provide a rich description of the context of the study to allow for readers to consider the extent to which the results are transferable to other settings, and we do not consider the results to be directly generalisable as genetic counselling practices differs. 

Finally, it is always a good idea to involve patients, here also ARRs representatives, in developing healthcare working models, an obvious but sometimes neglected aspect [29].

The interviews in this study were performed by four persons. While this could complicate the interpretation of data, the strategy also prevented close personal connections between the interviewer and the participant. We evaluated the sample size and adequacy using the concept of information power described by Malterud et al. [30]. Fourteen participants were included in the study; this sample size was considered appropriate. A possible limitation was the observation that some non-genetic HCPs had less experience of the concept of risk disclosure which could equal a sparser specificity, on the other hand the diversity among participants is a strength since we gained a wide range of attitudes.

It was outside the scope of the study to analyse for tendencies to take a certain position based on the participants’ profession and therefore only occasionally commented. Participants included 13 women and one man which in part represents a female domination among HCPs performing genetic counselling.

## Conclusion

We present four positions that healthcare professionals have, take, and move between, representing their attitudes towards counselling patients about risk disclosure to at-risk relatives. Genetic testing is both treatment-informing and a family matter, with several ‘characters in play’. With changing practices in the mainstream testing era comes a need for inter-professional dialogue about when and how to provide adequate genetic counselling, including the issue of responsibility for at-risk relatives. We hope that these results can serve as a basis for discussions on these roles and responsibilities, moving best practices forward.

## Supplementary Information

Below is the link to the electronic supplementary material.

Supplementary file1 (DOCX 14 KB)

## Data Availability

The data that support the findings of this study are not openly available due to reasons of sensitivity and are available from the corresponding author upon reasonable request. Data are located in controlled access data storage at Umeå university.
