# Supplementary material for: “Choosing the main character”: healthcare professionals’ attitudes towards counselling patients about risk disclosure to relatives in the era of mainstream cancer genetic testing
Source: Fam Cancer. 2025 Dec 12;25(1):3. doi: 10.1007/s10689-025-00516-1 (PMC12700921; doi:10.1007/s10689-025-00516-1)
Supplement: Supplementary file 1 — Supplementary file1 (DOCX 14 KB) [file 10689_2025_516_MOESM1_ESM.docx]

## Supplementary info

**Interview guide,** last version, May 2024

When speaking with the patient about sharing information to their family, how do you usually do it?

Why then? What are your thoughts? What usually happens? How do patients react to it?

Do you have any specific strategies or approaches?

When do you find it difficult? What are your thoughts? What do you do?

In a patient meeting, how much of the conversation is dedicated to discussing information spreading?

Do you follow up in any way to ensure the patient understands what information to share and how to share it?

Have you ever thought about wanting to work differently? Different approach? Why is that?

If we talk more generally about heredity and whether the family receives information, do you have any thoughts on that?

What do you think about the patient's role?

What do you think about your own role?

What are your thoughts on the patient's responsibility versus yours and the healthcare system's responsibility?

What are the advantages and disadvantages of the patient's responsibility versus the healthcare system's responsibility?

Do you ever wonder if information will be shared? How do you handle that if so?

Do you feel responsible for anyone other than the patient, i.e. ensuring family members are informed?

Genetic testing of cancer patients is becoming more common. New approaches are being developed, such as non-geneticists providing limited information, verbally and in writing, before genetic analysis is done on a tumour or blood sample. One might think this affects the healthcare system's responsibility, or not. Do you have any reflections on this?

If your relative had cancer and a genetic cancer risk, how would you want the information to be shared?
